# Characterizing the Mechanical Behavior of Bone and Bone Surrogates in Compression Using pQCT

**DOI:** 10.3390/ma15145065

**Published:** 2022-07-20

**Authors:** Johannes D. Pallua, David Putzer, Elias Jäger, Gerald Degenhart, Rohit Arora, Werner Schmölz

**Affiliations:** 1 Department of Orthopaedic and Trauma Surgery, Medical University of Innsbruck, 6020 Innsbruck, Austria; johannes.pallua@i-med.ac.at (J.D.P.); david.putzer@i-med.ac.at (D.P.); elias.jaeger@hotmail.de (E.J.); rohit.arora@i-med.ac.at (R.A.); 2 Department of Radiology, Medical University of Innsbruck, 6020 Innsbruck, Austria; gerald.degenhart@i-med.ac.at

**Keywords:** micro-compression, micro-tomographic imaging, pQCT, microstructural bone failure, bone architecture

## Abstract

Many axial and appendicular skeleton bones are subjected to repetitive loading during daily activities. Until recently, the structural analysis of fractures has been limited to 2D sections, and the dynamic assessment of fracture progression has not been possible. The structural failure was analyzed using step-wise micro-compression combined with time-lapsed micro-computed tomographic imaging. The structural failure was investigated in four different sample materials (two different bone surrogates, lumbar vertebral bodies from bovine and red deer). The samples were loaded in different force steps based on uniaxial compression tests. The micro-tomography images were used to create three-dimensional models from which various parameters were calculated that provide information about the structure and density of the samples. By superimposing two 3D images and calculating the different surfaces, it was possible to precisely analyze which trabeculae failed in which area and under which load. According to the current state of the art, bone mineral density is usually used as a value for bone quality, but the question can be raised as to whether other values such as trabecular structure, damage accumulation, and bone mineralization can predict structural competence better than bone mineral density alone.

## 1. Introduction

The mechanical properties of bone depend on its structural and material organization, primarily attributed to its composition, which defines the types of loading it can endure [1]. Across different bone types, species, and levels of hierarchical organization, the tissue’s structural properties are influenced by its architectural arrangement [2,3]. The amount of loading a bone can sustain before fracture is determined by its architectural configuration [4,5]. The trabecular bone should be considered when it comes to the structure of bones at the tissue level. The trabecular bone is composed of trabecular rods and struts, which provide large marrow-encapsulating spaces [6]. An important load-bearing and protective structure of the body is the cortex. The material is mainly resistant to compressive deformations while remaining relatively weak in tension [7]. Furthermore, microstructural porosity significantly influences bone’s mechanical properties [5,8]. Several studies have identified microdamage as a critical factor affecting bone mechanics [9].

Bones in the axial and appendicular skeleton undergo repetitive loading during normal daily activities. If this load increases sufficiently over a prolonged period, it can lead to the failure of the bone [10]. A dynamic loading process with simultaneous image-guided recording can be used to analyze the values at which tissue deformation begins. For this purpose, an image-guided technique for analyzing structural defects using step-wise micro compression in combination with time-delayed micro-tomographic imaging is being developed. At a microscopic level, fracture initiation and progression will be directly visualized and quantified in three dimensions.

Moreover, this technique can be used to diagnose the local failure pattern of bones. Bone stiffness helps assess bone quality and orthopaedic design prostheses, among other clinical applications. During the in vitro testing of bone, specimens are commonly conducted using compression testing, an experimental technique [11]. The stiffness of structures or materials is defined by their resistance to deformation [12]. In understanding the relationship between the structure and function of bone, this property is crucial and has clinical relevance in orthopaedic prosthesis design and characterization of bone properties via anatomical sites [13,14,15,16]. In order to understand the effects of a disease, age, and medical interventions on bone, physicians must accurately and efficiently measure bone stiffness.

The relationship between bone microstructure and the behavior of microdamage in bone has been described and quantified in several studies [17,18,19,20,21]. Small amounts of microdamage in bone led to significant reductions in bone strength and stiffness [22]. Despite this, it is still not fully understood where the accumulated microdamage is located nor how the bones will fracture. It has been challenging to study the dynamic process of bone fracture due to the lack of temporal resolution in the link between bone structure and function.

Microdamage initiation and propagation can be visualized using both two-dimensional (2D) imaging methods, such as histology and microscopy [23,24,25,26], and three-dimensional (3D) image-based methods, such as confocal microscopy [27,28] and computed tomography (CT) [29,30,31,32].

CT systems include medical CT, high-resolution quantitative CT, and micro-CT (µCT) to provide bone images non-invasively at different levels of structural details ranging from macro to micro. Medical CT and µCT provide 3D images, allowing more desirable analyses than 2D X-ray images. These CTs have become highly popular in bone research as they provide both density and structure data for bone tissue. Over the past decade, several imaging techniques have been utilized with increasing capabilities and image resolutions. Major work in bone structure has been carried out using various methods reporting trabecular structure in specimens from human, cadavers, and animal models [33,34]. The synchrotron technology used in µCT was able to acquire detailed features of trabecular bone architecture down to a resolution up to 0.5 µm [35]. However, up to date synchrotron imaging technology is limited to preclinical research and cannot be applied in clinical practice due to the construction and maintainance costs, building time of accelerators, use of high-radiation sources, maintenance of high-security zones, generation of big data, operation by specialists, as well as limitations on its use in preclinical or clinical research [36].

Image-guided failure assessment (IGFA) has been introduced in conjunction with CT through various in situ testing systems with varying degrees of automation [37,38,39]. However, the loading devices required manual operation [40] or were limited by their geometric design [41,42] or mechanical capabilities [37]. Moreover, only one of the above-listed loading devices has been validated for precision and accuracy [37]. Because IGFA is a promising method to assess dynamic failure processes, the current work is a proof of concept demonstration aimed at developing a novel micro-mechanical test system combining the compression of bone cylinders of cancellous bone material and simultaneous imaging, with simultaneous imaging as a peripheral quantitative computed tomography (pQCT). For this purpose, sample cylinders made of different materials, bone surrogates (Sawbone), and lumbar vertebral bodies from bovine and red deer were embedded and loaded step-wise. Consequently, the test system is validated to perform step-wise load testing of trabecular bone specimens based on image-guided displacement analysis.

## 2. Materials and Methods

**Specimen preparation:** Two bone surrogate groups consisting of urethanes, epoxies, and structural fillers were used (Manufacturer: Sawbones Europe AB, Malmö, Sweden, Product Code: 1522-626-01, 1522-525). The density and loading characteristics of the bone surrogates are presented in Table 1.

The biological samples were six lumbar vertebral bodies, three from bovine (*Bos taurus*, female, 18 months) and three from red deer (*Cervus elaphus*, male, 3 years). Cylindrical specimens (Ø 15 mm × height 25 mm) were manufactured using a core drill, and a miter saw. In setting up the investigation, we followed the experiment procedure of Nazarian and Muller, 2004 [37].

**Load levels definition:** Specific loading steps were determined for the different sample types based on their density properties to recognize the course of the trabecular change in the later pQCT scans. For this purpose, one specimen of each group (*Bos taurus*, *Cervus elaphus*, SB 525, SB 526) was subjected to a load to failure test in a uniaxial testing machine (MTS, 858 MiniBionix II, Eden Prairie, MN, USA). By recording the force and the axial displacement stress–strain curves, five loading steps (including preloading, 50 N) were determined for each of the subsequent micro-compression tests in the pQCT. The loading steps were selected in the following way: at 50 N was the preloading of all experiments, two load steps were selected within the elastic phase of the material, one loading step each was selected before and after reaching the ultimate strength of the samples.

**Figure 1:**
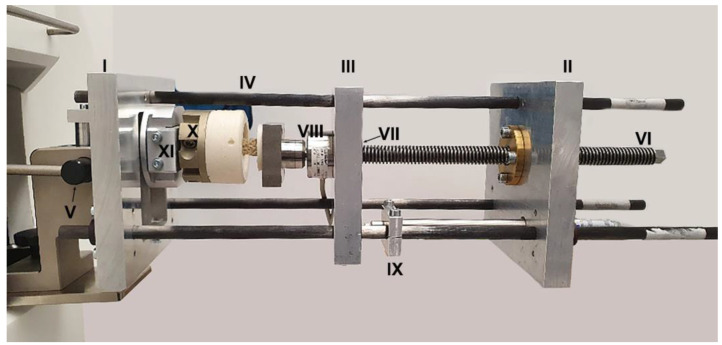
The compression device: The basic structure consists of three steel plates; front, rear, and center plate (I, II, III). The front and rear plates are connected by three cylindrical carbon rods (IV). Reference ball pivots (V) are attached to the front and rear plates, from which the compression device can be suspended in the XtremeCT II. The ball pivots represent the only contact surface to the XtremeCT II. The center plate is suspended on the carbon rods, which can move freely along the rods. A threaded spindle (VI) fixed via a spindle nut (VII) on the center plate exercises compression load on the specimen to adjust the compression intensity. The cylindrical block of embedding material was screwed to a flange (X), which is attached in the compression device to a mounting mechanism (XI). Force is measured by the load cell (VIII) and displacement by the LVDT sensor (IX).

**Micro-compression tests:** Step-wise micro-compression combined with time-lapsed micro-computed tomographic imaging was performed using a compression device suspended from the XtremeCT via ball pivots (Figure 1). To step-wise load, the cylindrical specimens under uniaxial loading conditions in the clinical pQCT XtremeCT II (Scanco Mecial AG, Brüttisellen, Switzerland), a guidance system (Figure 1) was used. A load cell (KAM, maximum load 5 kN, precision 0.2 N, Angewandte System Technik GmbH, Dresden, Germany) (Figure 1. VIII) was centrally mounted on the center plate. Force displacement curves were recorded using an LVDT sensor (WTA20LX/M, Peekel Instruments GmbH, Bochum, Germany) and the load cell. The load cells were connected to the amplifier system and calibrated. The specimen was placed in the cylindrical block of embedding material and loaded with a preload of 50 N. The cylindrical specimen formed an axis with the threaded spindle. Subsequently, the load model and the clamped sample were pushed into the XtremeCT, and the first scan was performed. Once the scan was complete, the load was increased using a threaded spindle to the defined axial load using the PICAS display (PICAS, PEEKEL Instruments, Bochum, Germany) for verification. This process was repeated until the trabecular structure of the sample yielded. Stress and strain were calculated.

The clinical pQCT XtremeCT II was used to visualize the change in the trabecular structure during compression. The parameters in pQCT were developed based on the assumption that the mechanical properties of the trabecular structure depend not only on the density of the material but also on the trabecular network [43]. Pre-settings of all scans included a resolution of 60, 7µm Isovoxels, an integration time of 46 ms per projection, and a voltage and intensity of 68 kV/1410 µA based on the standard patient scanning settings. Radiation dose for one scan ranged between 0.009–0.018 mSv.

**Data analysis:** Regarding the post-processing, the focus was on creating 3D images of the pQCT scans using the internal system workstation, which includes a multiprocessing virtual memory-based operating system (VMS) (Hewlett–Packard, Palo Alto, CA, USA) in combination with an internal processing language (IPL) (Scanco Medical AG, Brüttisellen, Switzerland). For this purpose, the desired volume of interest (VOI) was selected layer by layer from the pQCT scans, and 3D images were reconstructed. The process calculates various parameters from the VOI that can be used to compare bone mineral density (BMD) and trabecular structure. The meaning of the parameters is presented in Table 2.

**Figure 2:**
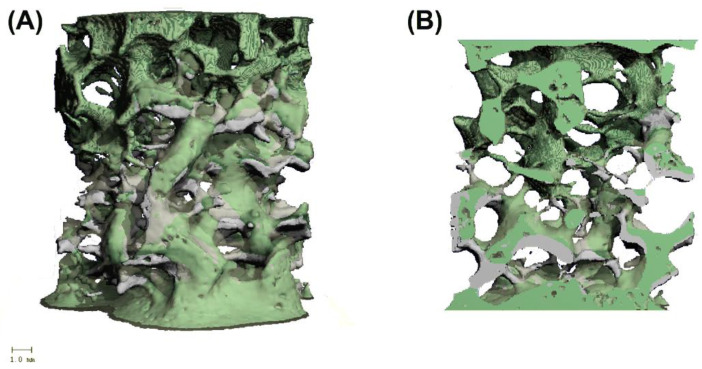
Overlay of two 3D Images, acquired from step-wise testing of an SB 526 specimen at 50 N (magenta) and 300 N (gray); (**A**) front view, (**B**) inside view.

By superimposing two 3D images of the same test cylinder with different loads, structural changes in the trabeculae were visualized. Thus, it can be visually understood at which load and at which point the trabecular bone material changes or fails. Horizontal 2D slices were calculated from the superimposed 3D images to determine the exact trabecular structure change or failure. The surfaces of the basic image, the second image, and the superimposed area within these slices can be selected and compared with other samples. The sectional images of the superimposition were exported as DICOM files and processed with MATLAB (Version 9.9, R2020b, The Mathworks Inc., Natick, MA, USA ). Using MATLAB, the area fractions were calculated, and the pixel values of the images were adjusted to display them visibly. The aim of overlaying two 3D images was to show the changes of the trabecular bone structure in 3D space on the one hand, and to use horizontal layers of these images on to determine the areas of the overlay and the superimposed area on the other hand. Since a 3D image was calculated from the pQCT scans for each load step, all levels can be compared with the initial sample cylinder. This, in turn, provides precise insight into the structural change of the sample trabeculae at each loading step, like in Figure 2, where an overlay of two 3D images of a specimen cylinder (SB 526) is shown.

**Figure 3:**
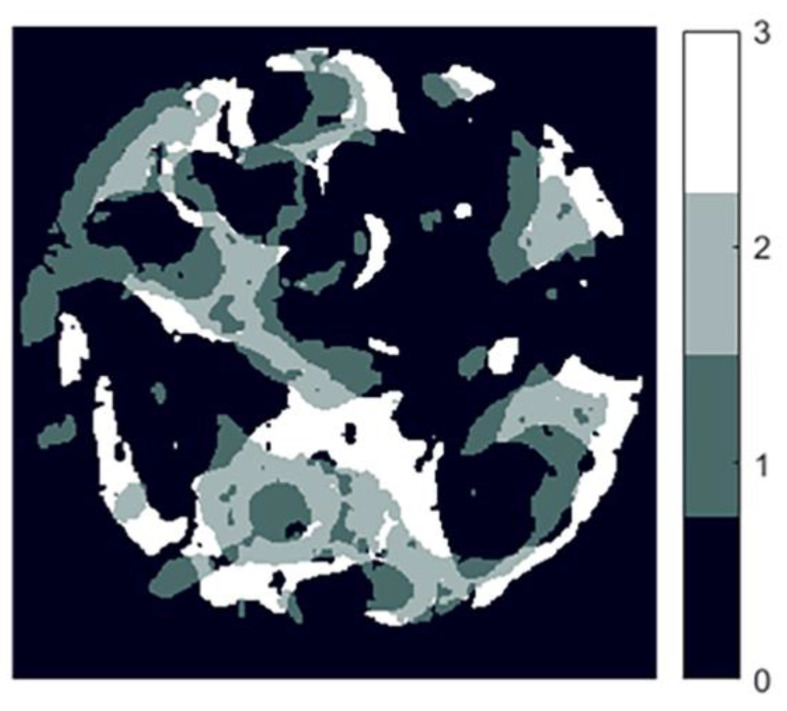
Layers of the superimposed 3D images indices and correlated colors of the different regions; 0 background, 1 sample 3D image, 2 overlay area, 3 initial.

In order to make a precise statement about the structural change of the trabeculae via the increasing force, the superimposed 3D images must be split into horizontal 2D slices (Figure 3). The number of layers was based on the size of the 3D image. At the ends of the sample cylinders, the superimposition of two 3D images of different loads does not usually provide any information about the change or failure of the trabecular structure since the more heavily loaded sample was always compressed and was thus smaller than the initial sample. Therefore, the layers without significance had to be filtered out during the evaluation.

3D images were created using MIMICS segmentation software (Mimics V16.0, Materialize, Leiden, Belgium) using a threshold function for bones between 1250 to 4095 Houndfields Unit. Mean, and standard deviation was calculated for the various measurement parameters using Scanco Software. Excel (Microsoft Office Professional Plus 2010, Redmond, WA, USA) was used for further analysis and graphs.

## 3. Results

The main focus of this study was the evaluation of a micro compression device for microstructural imaging of bone failure behavior in a trabecular bone specimen using pQCT.

### 3.1. Load Levels Definition

Concerning the load to failure tests of the specimens, all types show different maximum forces (LOF) at the fracture of the trabecular structure. The SB 525 sample withstood a load of 640 N. Besides the load steps of 50 N, the two load steps within the elastic phase were chosen to be 400 N and 500 N, the two load steps around the LOF were selected to be 600 N and 700 N. The SB 526 sample demonstrates a LOF of 360 N and the respective load levels were chosen to be 50 N, 200 N, and 250 N in the elastic phase and 300 N and 400 N around the LOF. The *Bos taurus* specimen fractured at a load of 1758 N. The load levels were chosen to be 50 N, 1000 N, and 1500 N in the elastic phase and 1750 N and 1800 N around the LOF. LOF occurred in the *Cervus elaphus* samples at an axial force of 4767 N. The load levels were chosen to be 50 N, 1000 N, and 1500 N in the elastic phase, while 1750 N and 2000 N were near the LOF point. This test had to be stopped as it reached the force limit. An increase over 2000 N was not possible due to the design of the compression device for the micro-compression tests (e.g., the small diameter of the carbon rods, bending of the linkage). In Table 3, the LOF and the load steps of all samples are listed.

**Figure 4:**
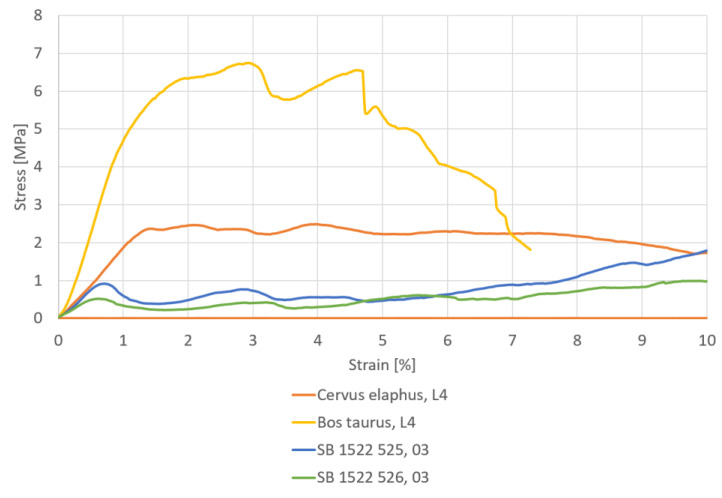
Stress–strain curves for the four samples determined in the uniaxial compression test.

The stress–strain behavior of the samples is shown in Figure 4. Both bone surrogates show different characteristics from that the biological samples. The curve decreases after a linearly increasing phase up to the ultimate strength. An ultimate strength of 0.5 MPa was determined for the bone surrogate SB 1522 525 and 0.9 MPa for SB 1522 526. The determined ultimate strength was 2.5 MPa for the *Bos taurus* L4 and 6.7 MPa for the *Cervus elaphus* L4.

### 3.2. Micro-Compression Tests

The bone surrogate cylinder samples showed significantly lower failure loads than the biological samples. Both specimens consisting of the 1522-526-01 foam failed at 300 N. The two SB 526 samples withstood a load of 550 N and 600 N. During the load tests in the XtremeCT II, the *Cervus elaphus* bones (two samples) withstood up to the last load step (2000 N). No direct fracture progression could be detected on the pQCT scans. However, the trabecular structure showed small changes near the embedded sites. The current design of the compression device is not capable of applying higher loads than 2000 N due to device distorsion. The bone structure of the bovine specimens macroscopically fractured at 1000 N and at 1500 N.

**Figure 5:**
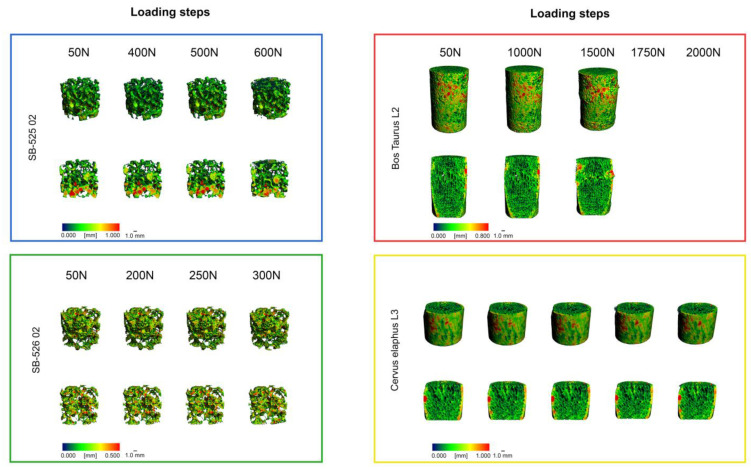
3D rendering of pQCT scans of each specimen dataset without and with cross-section for spatial thickness distribution (0.000–0.800 mm).

**Figure 6:**
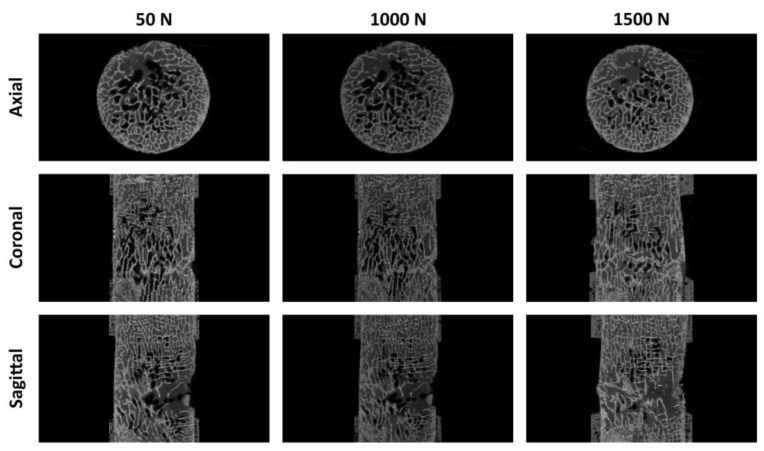
Axial, coronal and sagittal images of *Bos taurus* L2 sample 50 N and 1000 N, 50 N and 1500 N). The bone structure of the bovine specimens fractured at 1000 N and 1500 N.

If all specimens had endured to maximum loading, there would be five pQCT scans of each sample. However, since SB 525 (four scans), SB 526 (four scans), and *Bos Taurus* (three scans) cylinder samples failed at one of the specified loading steps, the number of scans available is not the same. The trabecular structural change can be analyzed well when comparing the preloaded (50 N) with the failure load. Each intermediate step gives more detailed information about the structural change over the increasing load. Figure 5 depicts all 3D models of the biomechanical samples and the bone surrogates. In Figure 6, axial, coronal, and sagittal images of *Bos taurus* L2 sample (50 N and 1000 N, 50 N and 1500 N) illustrate the bone structure of the bovine specimens fractured at 1000 N and 1500 N.

The most important structural parameters calculated by the pQCT software from the scans are shown in Table 4. The bone surrogates showed the highest values of BV/TV, Conn.D, SMI, Tb.N, and the lowest values of Tb.Sp at the failure load (SB 525, 600 N, and SB 526 300 N) compared to the other load levels. The mean value and the standard deviation of the samples are shown in each case. The ratio of bone volume to total volume was around 40% for the *Cervus elaphus* specimens at all loading levels and approximately 35% for the *Bos taurus* at all loading levels. The structural parameters Conn.D and Tb.N were the lowest for the *Cervus elaphus* specimens, and the structural parameters SMI and Tb.Sp was the highest compared to the rest of the loading levels. In terms of the bone mineral density, the biological samples did not show any immediate change between the load levels, and the bone surrogates showed a slightly lower density at the highest load levels.

### 3.3. 2D Cross-Sections of Boen Surrogates

**Figure 7:**
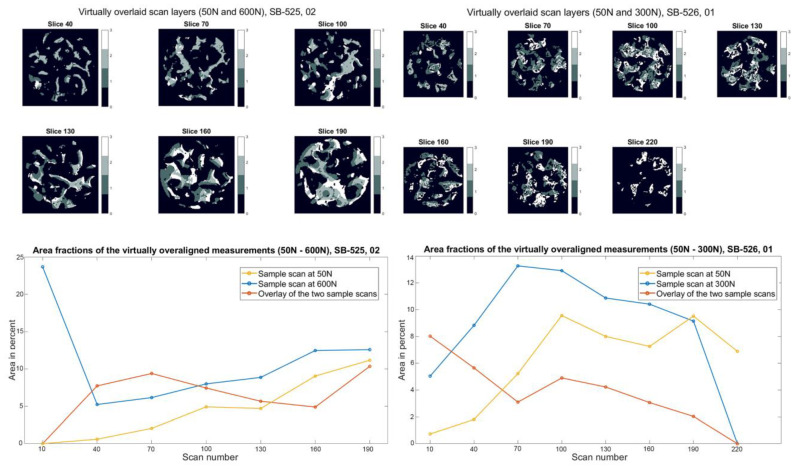
Slice view of the virtually superimposed 3D images (SB 525: 50 N and 600 N, SB 526: 50 N and 300 N). The different components are declared in each layer with numbers from 0–3 and the corresponding color. 0 Background, 1 Sample image (50 N/600 N and 50 N/300 N), 2 Superimposed area of the two 3D images, 3 Initial images (50 N). The course of the area fractions of the sample image (50 N/600 N and 50 N/300 N), the source image (50 N), and the superimposed area over the different layers are summarized in a diagram.

The SB 525 samples failed at a load of 600 N (Figure 7). At the first loading step (400 N), they showed a high percentage of overlap in all layers (between 5% and 25%) with simultaneous low area fractions of the overlapped images (below 5%). Due to the smaller number of trabecular structures in the bone surrogates, the area fractions are smaller than those of the biological samples. In the next higher loading level (500 N), evident structural changes are visible in the upper layers in all layers. In the case of the failure load, apparent differences can be seen, whereby the overlay decreases significantly, especially in the higher layers, with a simultaneous increase in the area proportion of the two superimposed images compared to the previous load levels. SB 526 samples showed high overlay percentages (between 6% and 16%) at the first loading step (200 N) in all layers. The specimens showed evident changes in the trabecular structure at the failure load (300 N), clearly seen in the layer images and the correlating plots (Figure 7).

### 3.4. 2D Cross-Sections of Biological Samples

**Figure 8:**
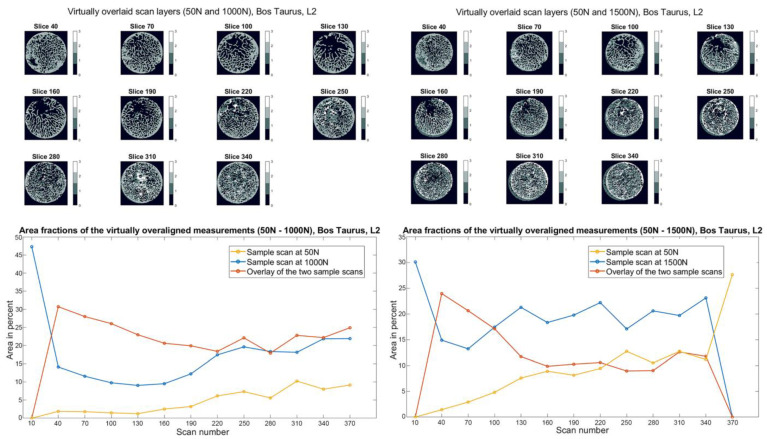
Slice view of the virtually superimposed 3D images (50 N and 1000 N, 50 N and 1500 N) from the *Bos taurus* L2 sample (**top**). The different components are declared in each layer with numbers from 0–3 and the corresponding color. 0 Background, 1 Sample image (1000 N/1500 N), 2 Superimposed area of the two 3D images, 3 Initial image (50 N). The course of the area fractions of the sample image (1000 N/1500 N), source image (50 N), and the superimposed area over the different layers are summarized in a diagram.

The superimposed scan layers and the corresponding diagram of the *Bos taurus* sample L2 in Figure 8 show that the overlay’s area portion is dominant (25–30%) in layers 40–100. This means that the 3D images are similar in this area, and no recognizable structural change of the trabeculae has occurred. In the higher layers (starting from 130), the area of the overlay decreases, which means that the 3D images are no longer the same, and structural changes have occurred in this area due to the load (1000 N). This behavior can be seen in the diagram, whereby the overlay in the lower layers is most significant at the beginning and decreases as the layer number increases. At a load of 1500 N, the bone structure failed. The failure can be seen in the superimposed sectional images and the corresponding diagram in Figure 8. At layers 40–70, the overlay is no longer present to the same extent as at the previous loading step. The area fraction has dropped below 25% at layer 40 and decreases further with increasing layers to a percentage value of almost 18.

**Figure 9:**
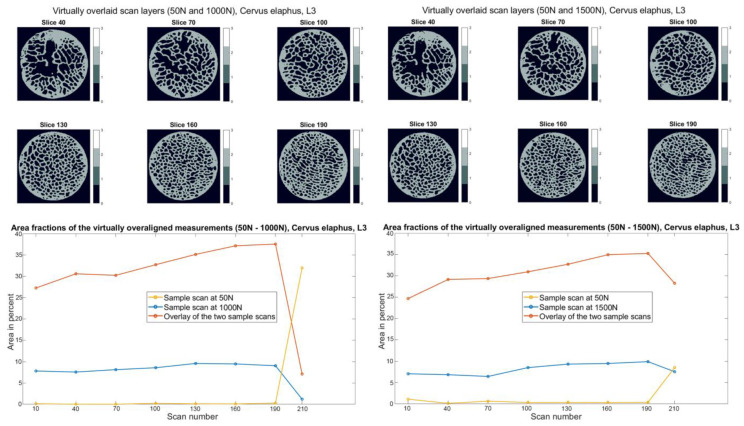
Slice view of the virtually superimposed 3D images (50 N and 1000 N, 50 N and 1500 N) from the *Cervus elaphus* L2 sample (**top**). The different components are declared in each layer with numbers from 0–3 and the corresponding color. 0 Background, 1 Sample image (1000 N/1500 N), 2 Superimposed areas of the two 3D images, 3 Initial images (50 N). The course of the area fractions of the sample image (1000 N/1500 N), source image (50 N), and the superimposed area over the different layers are summarized in a diagram.

The cross-sectional images of the superposition of the *Cervus elaphus* specimen L2 output image (3D image at 50 N) with the 3D image of the specimen at 1000 N and 1500 N are shown, with their corresponding plots in Figure 9. They offer very little to no structural change in the trabecular structure of the bone material. The area fraction of the superimposition is between 15% and 30%, with shallow area fractions of the superimposed 3D images.

## 4. Discussion

From a macroscopic to microscopic perspective, it is essential to understand the hierarchy of events in bone [44,45]. Visualization and analytical techniques make an assessment at each level feasible, but a single sample cannot be subjected to the full range of mechanical testing techniques because of their destructive nature. The mechanical examination is often combined with visualization techniques [1]. In the present proof of concept study, micro-compression experiments and IGFA were used to visualize and study the initiation and propagation of microdamage on macroscopic and microscopic scales. Similar devices combining mechanical compression with CT imaging of bone failure have been reported in other studies [1,37,40,41,42,46]. Still, those experimental setups had limitations, such as the loading devices either requiring manual operation [40], being limited by their geometric arrangements [41,42], or mechanical capabilities and validation [37]. The circumvention of the mentioned limitations involved constructing and incorporating a micro-compression device at a pQCT. pQCT is currently the highest resolution in vivo scanner available for clinical human bone measurements. It is imperative to demonstrate the utility of this cutting-edge research tool before it can be used in routine clinical practice to predict fractures. Despite being strictly a research tool today, pQCToffers the possibility of broader use in the hands of non-experts when used in the standard analysis or automated analysis default mode. When pQCT is proven to be beneficial to bone quality assessment, the currently prohibitive cost of the systems will likely decline as production increases. As normative databases are developed, it will be possible to provide a better context for the outcome measures of pQCT, similar to the T-scores that are routinely reported from DXA scans [47].

Both ex vivo and in vivo imaging of bone tissue have progressed toward providing high-resolution images. The improved image quality, analysis, and diagnostic capabilities of pQCT [48,49,50] have advanced research in BMD measurement, implant interface [51], and cartilage research [52,53]. Regarding submicron resolution imaging, the ex vivo application has greatly improved spatial resolution for larger specimens and decreased scan times. Clinical and research imaging technologies will continue to improve and help understand microdamage behavior in bone [35].

Focusing on the structural parameters in Table 4, it is noticeable that the BMD values differ only slightly, and a clear statement as to whether changes in the trabecular structure up to fractures occur is hardly impossible. It should be mentioned that the small number of samples and the associated high standard deviation makes it very difficult to clarify changes concerning all parameters. Nevertheless, pQCT-specific parameters have shown small changes in selected samples, especially at the highest loading level. The connectivity density of the trabeculae and the fraction of bone volume increases, especially for the bone surrogates, at higher loadings. This can be explained by the trabeculae being pressed into each other, meaning that the Tb.N becomes smaller, and the BV increases. Another significant value is the SMI, which describes the shape of the trabeculae. It clearly shows the different trabecular shapes of the biological samples (values between 0.84 and −1.759), which instead indicate concave shapes (<0), compared to the bone surrogates, which instead show a mixture of planar and cylindrical shapes (values between 0.798 and 1.865). Here, the mean ellipsoid factor (EF), fitting ellipsoids in 3D space, could give better information as described in the work of Salmon [54]. The trabecular shape difference is also evident when comparing the 3D models and their sectional views with biological samples. The bone surrogates have significantly larger trabecular distances (Tb.Sp 1.762–1.986) than the biological ones (Tb.Sp 0.279–0.366), resulting in the biological specimens withstanding significantly more strain. In connection with the load, the samples of the *Cervus elaphus* material again withstood substantially more strain than the *Bos taurus* samples, which is possibly because wild animals are exposed to higher external loads in everyday life than bovines.

The morphology of their vertebral bodies is more prominent, which may better distribute the weight force. The evaluation of the load tests has shown that the structural parameters generated by the pQCT software describe the properties of the investigated bone material. In addition, the combination of the micro-compression test with subsequent pQCT scans can also provide information about the behavior of trabecular bone material under step-wise increasing load. Comparing the micro-compression device for dynamic image-guided failure assessment [1] with the test system developed in this work, the advantage of using the compression device designed for step-wise loading and the pQCT is clear.

On the one hand, a 3D model can be reconstructed from each scanned sample, in which any cross-section can be selected to investigate the exact trabecular structures. On the other hand, the samples can be loaded with axial forces of up to 2000 N. In this case, improving the mechanical properties of the compression device, especially the stiffness and resistance to distortion, could help. Concerning other techniques that have investigated local bone changes under load, the work of [37] provides helpful comparative literature. The authors loaded whalebone specimens and aluminium foams to failure loading and used a µCT for image processing. 

In contrast to the specimens used (diameter 15 mm, length 25 mm) in the present work, they used smaller cylindrical bone specimens (diameter 8 mm, length 16 mm), which they loaded to ultimate strength 16.185 MPa (whale specimens) and 2.108 MP an (aluminium foam specimens with the highest density). With the cross-section of the specimens, these values correspond to an axial force of 813 N for whale samples and 105 N for the aluminium foam samples. For the cross-section of the specimens used in the present study, the reported ultimate strength would correspond to an axial load of 2860.13 N for the whale specimens and 372.51 N for the aluminium specimens. Therefore, there are similarities in LOF, with the whale samples between the biological samples in this work and the aluminium foam with the highest density having almost the same ultimate loading as the SB 526 samples. The result of Nazarian et al. [37] also shows that the bone material’s quality and the associated trabecular geometry may be better assessed if, in addition to bone density, the structural composition of the bone, the accumulation of fractures, and bone mineralization are also taken into account. Hence, the limitations of our study were the design of the compression device and the small number of samples. The compression device would have to be redesigned and reinforced to allow loading beyond 2000 N with more and smaller load steps. Therefore, the failure of the *Cervus elaphus* specimen could not be investigated. An extension to human samples and methods such as X-ray computed tomography with digital volume correlation [55,56,57] and 4D computed tomography [58] would also be desirable.

## 5. Conclusions

The applied method of superimposing the generated 3D models showed significant advantages in localizing the change in trabecular structure induced by loading. It is possible to compare each layer of a 3D image with another one of the same samples subjected to an increased load and makes it possible to infer from which load the most significant change in trabecular structure occurred in which layer. This method showed that trabecular changes had already happened in some layers before the failure load in the bone surrogates.

In summary, the following points should be addressed for future research:

- The automization of data analysis by developing technical and scientific protocols.
- The creation of a larger data pool of different bone surrogates and different animal bones to enable the selection of the best suitable material for orthopaedic and trauma training and specific research questions.
- Future experiments should be evaluated automatically, and several smaller load levels should also be tested.
- Incorporating digital volume correlation, in situ X-ray computed tomography, and 4D computed tomography.

## Figures and Tables

**Table 1:** Density and load characteristics of the used foam.

Samples	Density ^1^			Compression	
	(pcf)	(g/cc)	Volume Fraction	Strength (MPa)	Modulus (MPa)
1522-526-01	20	0.32	0.21	1.3	105
1522-525	30	0.48	0.31	3.2	270

^1^ ASTM D1622.

**Table 2:** pQCT parameters with associated abbreviations, description, and unit.

Metric Measures	Abbreviation	Description	Standard Unit
Total volume	TV	Volume of the entire ROI	mm^3^
Bone volume	BV	Volume of the region segmented as bone	mm^3^
Bone volume ratio	BV/TV	Ratio of bone volume to total volume in the ROI	%
Trabecular separation	Tb.Sp	Mean distance between trabeculae	mm
Trabecular number	Tb.N	Mean number of trabeculae per unit length	mm
Structure model index	SMI	Measure of trabecular structure (0) for parallel plates and 3 for cylindrical rods	
Connectivity density	Conn.D	Extent of trabecular connectivity normalized by TV	mm^−3^
Degree of anisotropy	DA	Ratio between maximal and minimal radius of the mean intercept length elliposid	

**Table 3:** Load of failure of one specimen each and the resulting load.

Sample	LOF [N]	Load Steps [N]
SB 1522 525	640	50–400–500–600–700
SB 1522 526	360	50–200–250–300–400
*Bos taurus*, L4	1758	50–1000–1500–1750–1800
*Cervus elaphus*, L4	4767	50–1000–1500–1750–2000

**Table 4:** Structure parameters of *Bos taurus*, *Cervus elaphus* and Sawbones Samples in Comparison.

Sample	Load Steps [N]	BV/TV [%]	Conn.D	TRI-SMI	Tb.N [mm]	Tb.Th [mm]	Tb.Sp [mm]
*SB 525*	50	18.7 ± 1.3	0.43 ± 0.02	1.376 ± 0.256	0.583 ± 0.002	0.508 ± 0.020	1.762 ± 0.025
	400	18.6 ± 1.3	0.44 ± 0.01	1.436 ± 0.250	0.583 ± 0.003	0.509 ± 0.023	1.762 ± 0.024
	500	18.6 ± 1.5	0.44 ± 0.02	1.432 ± 0.270	0.586 ± 0.003	0.512 ± 0.025	1.760 ± 0.017
	600	19.0 ± 1.7	0.52 ± 0.11	1.865 ± 0.161	0.607 ± 0.016	0.500 ± 0.002	1.698 ± 0.028
*SB 526*	50	15.7 ± 0.4	1.21 ± 0.14	0.798 ± 0.060	0.535 ± 0.008	0.316 ± 0.005	1.986 ± 0.029
	200	15.7 ±0.4	1.21 ± 0.11	0.805 ± 0.051	0.537 ± 0.006	0.316 ± 0.004	1.974 ± 0.023
	250	15.8 ±0.4	1.24 ± 0.12	0.820 ± 0.032	0.541 ± 0.009	0.315 ± 0.004	1.963 ± 0.033
	300	16.2 ±0.4	1.25 ± 0.13	0.858 ± 0.048	0.565 ± 0.011	0.315 ± 0.004	1.881 ± 0.460
*Cervus elaphus*	50	41.2 ± 6.8	1.35 ± 043	−1.759 ± 0.688	1.508 ± 0.123	0.356 ± 0.054	0.598 ± 0.054
	1000	40.8 ± 6.8	1.35 ± 0.41	−1.718 ± 0.690	1.508 ± 0.122	0.360 ± 0.052	0.599 ± 0.052
	1500	40.8 ± 6.7	1.33 ± 0.41	−1.704 ± 0.700	1.500 ± 0.114	0.361 ± 0.052	0.601 ± 0.051
	1750	40.9 ± 6.6	1.31 ± 0.38	−1.681 ± 0.666	1.483 ± 0.100	0.363 ± 0.050	0.609 ± 0.043
	2000	41.6 ± 6.2	1.31 ± 0.39	−1.638 ± 0.691	1.429 ± 0.036	0.366 ± 0.048	0.639 ± 0.004
*Bos taurus*	50	35.3 ± 3.8	4.92 ± 0.29	0.284 ± 0.249	1.596 ± 0.020	0.279 ± 0.033	0.606 ± 0.003
	1000	36.1 ± 4.1	4.95 ± 0.10	0.232 ± 0.295	1.595 ± 0.004	0.280 ± 0.028	0.603 ± 0.006

## Data Availability

Data will be provided on request.
